# MicroRNA-100 is involved in shrimp immune response to white spot syndrome virus (WSSV) and *Vibrio alginolyticus* infection

**DOI:** 10.1038/srep42334

**Published:** 2017-02-09

**Authors:** Zhi Wang, Fei Zhu

**Affiliations:** 1 College of Animal Science and Technology, Zhejiang Agriculture and Forestry University, Hangzhou 311300, China

## Abstract

In this study, we discovered that shrimp miR-100 was up-regulated at 24 h after WSSV or *Vibrio alginolyticus* infection, confirming its participation in the innate immune system of shrimp. The anti-miRNA oligonucleotide (AMO-miR-100) was applied to inhibit the expression of miR-100. After AMO-miR-100 treatment, the shrimp was challenged with WSSV or *V. alginolyticus*. The knockdown of miR-100 expression decreased the mortality of WSSV-infected shrimp from 24 h to 72 h post-infection and enhanced the mortality of *V. alginolyticus*-infected shrimp significantly. The knockdown of miR-100 affected phenoloxidase (PO) activity, superoxide dismutase (SOD) activity and total hemocyte count (THC) after the infection with WSSV or *V. alginolyticus*, indicating a regulative role of miR-100 in the immune potential of shrimp in the response to WSSV or *V. alginolyticus* infection. The knockdown of miR-100 induced the apoptosis of shrimp hemocytes, and *V. alginolyticus* + AMO-miR-100 treatment caused more hemocyte apoptosis than *V. alginolyticus* treatment. The miR-100 influenced also the morphology of shrimp hemocytes and regulated the phagocytosis of WSSV or *V. alginolyticus*. Thus, we concluded that miR-100 may promote the anti-*Vibrio* immune response of shrimp through regulating apoptosis, phagocytosis and PO activity and affects the progression of WSSV infection at a certain level.

MicroRNAs (miRNAs) are small non-coding RNAs that function by base pairing with messenger RNAs and are involved in the regulation of almost every biological pathway^1^,^2^,^3^,^4^. MiRNAs have been demonstrated to play critical roles in tumorigenesis of many human cancers^5^,^6^. Recent research reports suggest that host miRNAs exert important functions in the response to causative agents of a numbers of viral infections, such as hepatitis B virus, hepatitis C virus, dengue virus, and human immunodeficiency virus (HIV)^7^,^8^,^9^,^10^,^11^,^12^. In a previous study, the crustacean viral pathogen white spot syndrome virus (WSSV) induced over-expression of host miR-100^13^,^14^. And we found that 55 shrimp miRNAs were differentially expressed in response to *V. alginolyticus* infection^15^.

MicroRNA-100 is exceedingly conservative, and its sequence is identical in model species, such as *Mus musculus, Danio rerio, Homo sapiens, Xenopus tropicalis, Apis mellifera*, and *Bombyx mori*. MicroRNA-100 (miR-100) has been confirmed to act as a tumor suppressor in various types of human cancer, including epithelial ovarian cancer, non-small cell lung cancer, colon cancer, hepatocellular carcinoma, glioblastoma, osteosarcoma, and colorectal cancer^16^,^17^,^18^,^19^,^20^,^21^,^22^. In an earlier investigation, the silencing of shrimp miR-100 expression caused an increase in the apoptotic activity of hemocytes and led to a further decrease in the number of viral genome copies in virus-infected shrimp^23^. Our previous study showed that miR-100 was over-expressed after *Vibrio alginolyticus (V. alginolyticus*) infection in shrimp^24^. Present in marine ecosystems, *Vibrio sp.* can infect not only invertebrates such as shrimps and crabs, but also vertebrates, including fishes and mammals. *Vibrio sp.* prefers hypersaline habitats, which makes Kuruma shrimp (*Marsupenaeus japonicus*), an important to humans species abundantly present in the ocean, the main victim of one of its main victims. WSSV is another lethal pathogen of Kuruma shrimps, and the infection caused by WSSV can lead to over 90% mortality in one week. In this study, we further explored the role of miR-100 in the innate immunity response of shrimp to WSSV or *Vibrio* infection.

## Results

**Figure 1:**
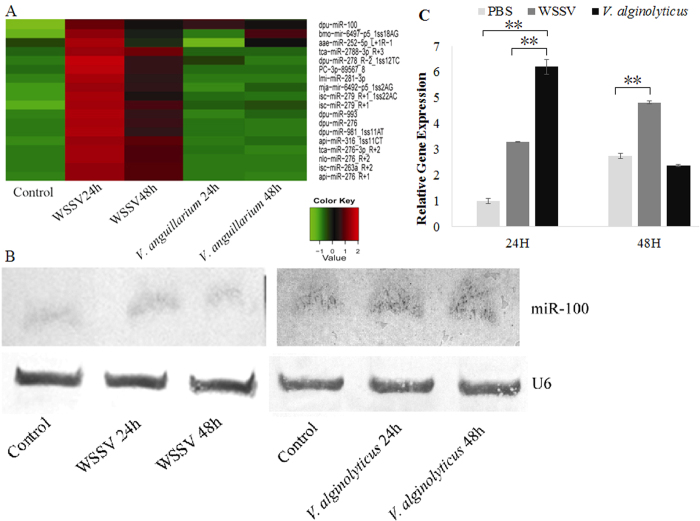
Identification of miRNAs response to WSSV or *V. alginolyticus* infection in shrimp through miRNA microarray and Northern blot. Total RNA was extracted from the hemocytes of un-infected or infected shrimp. (**A**) Hybridization of total RNA with miRNA microarray. The relative expression level of miRNA is shown with different colors; dpu-miR-100 indicates miR-100; (**B**) Northern blot analysis of total RNA with DIG-labeled oligodeoxynucleotide probes at different post-infection time points (24 and 48 h). (**C**) Quantitative Stem-Loop real-time PCR was also applied to reveal the post infection expression of miR-100 intuitively. The shrimp individuals in the control treatment were injected with saline solution, and U6 was used as a loading control.

Identification **of shrimp miRNAs response to WSSV or *V. alginolyticus***infection. Our results indicated that the expression of a number of miRNAs was significantly up-regulated 24 h after WSSV infection and down-regulated 24 h following *V. alginolyticus* infection (Fig. 1A). However, the data showed that miR-100 expression was significantly up-regulated 24 h after the infection with WSSV or *V. alginolyticus*. Then northern blot analysis and Quantitative Stem-Loop real-time PCR were performed to validate the findings of the miRNA expression profiles obtained by microarray assay. The results confirmed that the miR-100 expression was indeed up-regulated 24 h after the infection with WSSV or *V. alginolyticus* (Fig. 1B,C).

### Loss of function of shrimp miR-100 by using anti-miRNA oligonucleotide

**Figure 2:**
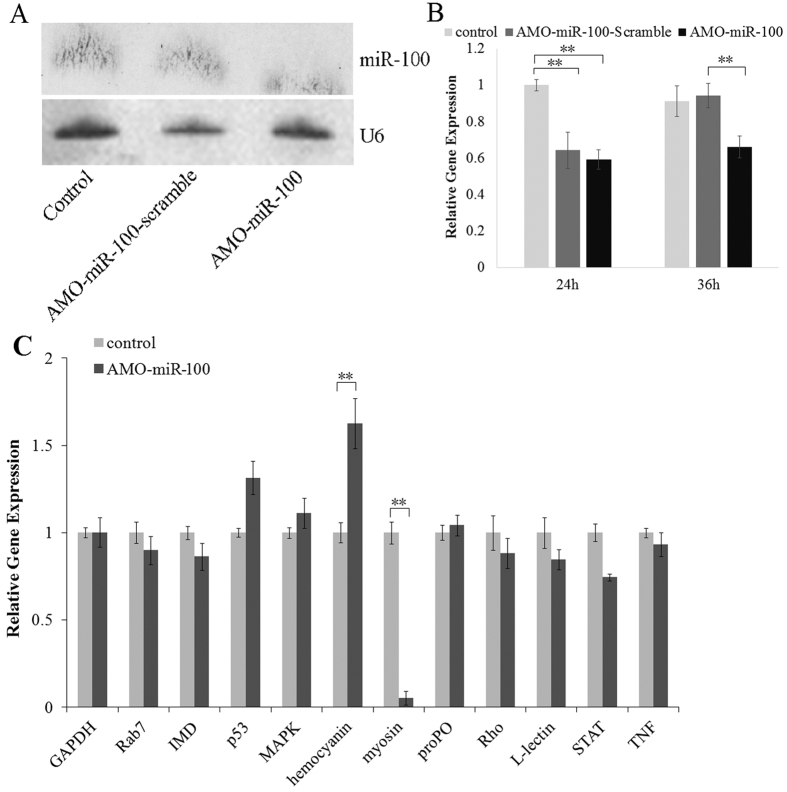
Loss of function of shrimp miR-100. Total RNA extracted from shrimp hemocytes at 24 h or 36 h after the AMO-miR-100 treatment were blotted with DIG-labeled oligodeoxynucleotide probes (**A**) and analyzed by Quantitative Stem-Loop real-time PCR to confirm the knock-down effect (**B**) and detect the expression in the hemocytes of eleven immune genes (Rab7, IMD, *p*53, MAPK, *hemocyanin, myosin, pro*PO, Rho, L-lectin, STAT, and TNF) (**C**). Uninfected shrimp individuals were used as negative control in Northern blot detection, and U6 was utilized as a loading control treatment. In real-time Q-PCR, the amount of target mRNA was normalized to the GAPDH transcript level. Data are shown as means ± SD (standard deviation) of three separate individuals. The asterisks indicate a significant difference (*P* < 0.01) between two samples.

To further investigate the role of miR-100 in the innate immunity of shrimp, a loss-of-function analysis was performed by silencing of miR-100 expression with anti-miRNA oligonucleotide (AMO-miR-100) (Fig. 2A,B). The results showed that the miR-100 expression was inhibited by AMO-miR-100, and was influence by the AMO-miR-100-scramble at 24 h but soon recovered at 36 h, indicating the specificity of AMO-miR-100. Fig. 2A showed the inhibition effect at 36 h. Then, we conducted real-time Q-PCR to analyze the expression of eleven known immune-related genes (*hemocyanin*, IMD, L-lectin, MAPK, *myosin, p*53, *pro*PO, Rab7, Rho, STAT and TNF). We choose these immune-related genes for their known function. Target genes and pathways of miRNA-100 were predicted by TargetScan and miRanda algorithms. The target gene of miRNA-100 is importin subunit alpha-3 and the pathway is protein transporter activity. This result makes no mean to our research, because we want to investigate the function of miRNA-100 only. The data revealed that *hemocyanin* was significantly (*P* < 0.01) up-regulated by the inhibition of miR-100, whereas *myosin* was significantly (*P* < 0.01) down-regulated (Fig. 2C).

### Effects of miR-100 knockdown on WSSV or *V. alginolyticus* infection

**Figure 3:**
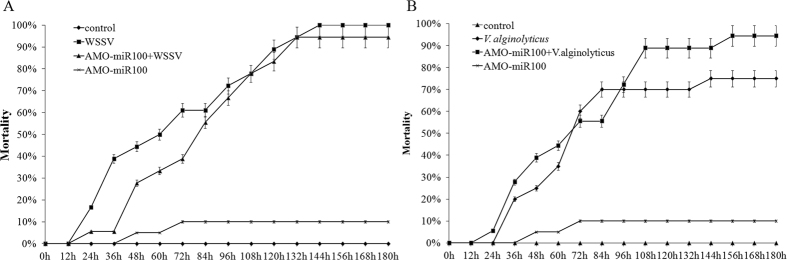
Mortality count of shrimp individuals challenged with WSSV (**A**) or *V. alginolyticus* (**B**) after the treatment with AMO-miR100. Each group contained over 20 shrimp individuals to ensure the confidence level and the treatment was repeated three times to avoid the influence of weather, shrimp condition, and injection operation error.

miR-100 was knocked down by AMO-miR-100, followed by WSSV challenge, to evaluate the effect of miR-100 on WSSV infection. The data showed that the treatment with AMO-miR-100 significantly decreased the mortality of WSSV-infected shrimp individuals and prolonged their lifespan from 24 h to 72 h post-infection when compared to that the life duration of the shrimp only challenged with WSSV (Fig. 3A). This phenomenon indicates that the knockdown of miR-100 expression inhibits the infection with WSSV to a certain extent. To investigate the effects of miR-100 knockdown on the mortality of *V. alginolyticus*-infected shrimp group, the shrimp group treated with AMO-miR-100 was challenged with *V. alginolyticus*. The results revealed that the AMO-miR-100 treatment significantly (*P* < 0.01) elevated the mortality of *V. alginolyticus*-infected shrimp when compared to that of the group with *V. alginolyticus* challenge only (Fig. 3B). These data indicate that miR-100 may contribute to the anti-*Vibrio* immune response of shrimp and affect the mortality of *V. alginolyticus*-infected shrimp.

### Influence of miR-100 knockdown on phenol oxidase (PO) activity

**Figure 4:**
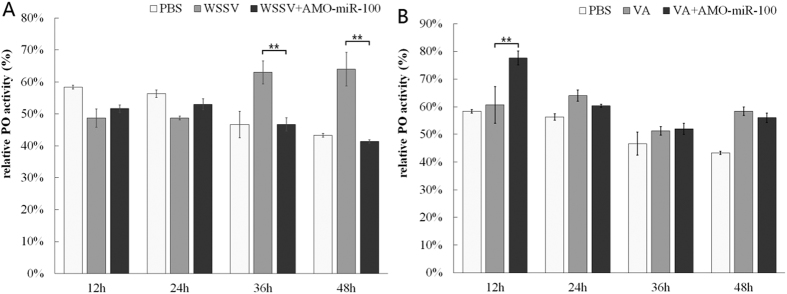
Effects of AMO-miR-100 interference on phenol oxidase (PO) activity. Shrimps were treated with a pathogen (WSSV or *V. alginolyticus*) only, with a combination of pathogen, and with AMO-miR-100 (WSSV + AMO-miR-100 or VA + AMO-miR-100). PBS was use as a control. VA stands for *V. alginolyticus*. (**A**) Hemocytic PO activity after WSSV or WSSV + AMO-miR-100 treatment; (**B**) Hemocytic PO activity after *V. alginolyticus* or *V. alginolyticus* + AMO-miR-100 treatment. Each column represents the mean of triplicate assays.

After WSSV infection, the PO activity was clearly increased and over 10% higher than that in the PBS control (Fig. 4A). It is noteworthy that no significant difference in PO activity was found between WSSV and WSSV + AMO-miR-100 groups in the first 24 h. However, the PO activity of WSSV + AMO-miR-100 group was significantly (*P* < 0.01) lower than that of the WSSV group 36 and 48 h post-treatment. This result showed that the knockdown of miR-100 weakened the PO activity after the WSSV infection, indicating that miR-100 can regulate the proPO system of shrimp in response to WSSV infection.

After the infection with *V. alginolyticus*, the PO activity in the infected group was slightly higher than that in the PBS control, but no significant fluctuation was observed as infection time increased (Fig. 4B). The PO activity of the *V. alginolyticus* + AMO-miR-100 group was significantly higher (*P* < 0.01) than that of the *V. alginolyticus* group at 12 h post-treatment. Within the interval from 24 h to 48 h post-treatment, the PO activity of the *V. alginolyticus* + AMO-miR-100 group deceased even further in comparison to that of the *V. alginolyticus* group. This result suggested that the knockdown of miR-100 affected PO activity after the infection with *V. alginolyticus*, indicating a regulative role of miR-100 in the proPO system of shrimp in response to *V. alginolyticus* infection.

### Influence of miR-100 knockdown on superoxide dismutase (SOD) activity

**Figure 5:**
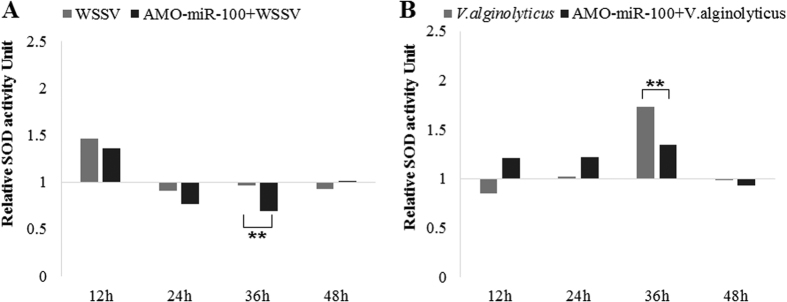
Effects of AMO-miR-100 interference on SOD activity of shrimp hemolymph. Shrimps were separated into different treatment group as in Fig. 4. PBS was use as a control. (**A**) Relative SOD activity after WSSV or AMO-miR-100 + WSSV treatment; (**B**) Relative SOD activity after *V. alginolyticus* or AMO-miR-100 + *V. alginolyticus* treatment. The SOD activity of PBS was used as an index as 1, each column represents the relative value compared to PBS.

After WSSV infection, the SOD activity of shrimp hemolymph was near 50% higher than control group at 12 h, at 24 h, the SOD activity dropped near 10% lower than PBS control group, and kept this trend to 48 h (Fig. 5A). As the expression of miR-100 was inhibited, the SOD enzyme was not as active as WSSV infected group, significantly lower than WSSV group or PBS group at 24 h, 36 h, then slowly recovered at 48 h. After *Vibrio. alginolyticus* infection, the SOD activity of shrimp hemolymph dropped below control at 12 h, then raise over 1.5 times higher than PBS group 24–36 h, declined again at 48 h (Fig. 5B). The SOD activity of shrimp hemolymph increased at 12 h, then as the knock-down effect of AMO-miR-100 started to work, at 36–48 h, the SOD activity smoothly increased, and showed a relatively lower SOD activity than that of *Vibrio* infected group. SOD activity of shrimp hemolymph was enhanced immediately after WSSV infection, but faded after 24 h, indicating the decrease of shrimp immunity. Inhibition of miR-100 weakened the SOD activity of shrimp hemolymph post WSSV infection immediately, indicating that the shrimp immunity was decreasing without miR-100. Meanwhile, after the Vibrio infection the SOD activity kept increasing in 12–36 h. In the lack of miR-100, the increase of SOD activity slowed down and was relatively lower at late stage. This result implied a positive role of miR-100 in shrimp immunity.

### Influence of miR-100 knockdown on total hemocyte count post infection

**Figure 6:**
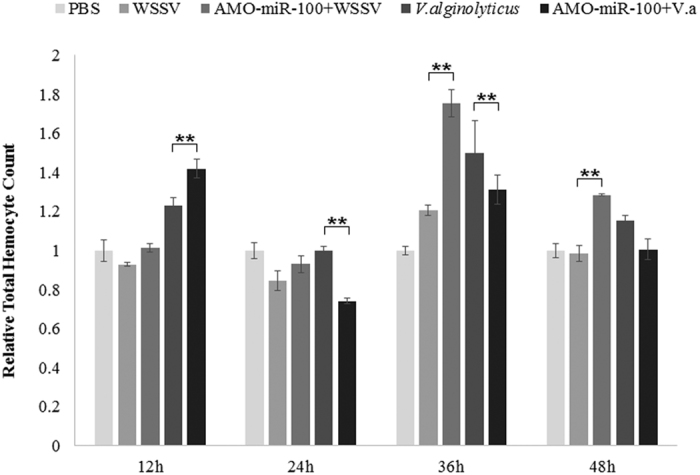
Effects of AMO-miR-100 interference on shrimp total hemocyte count. Shrimps were separated into different treatment group as in Fig. 4. PBS was use as a control. The total hemocyte count (THC) of PBS was used as an index as 1, each column represents the relative value compared to PBS. Each treatment contained at least three shrimps, and was counted for at least three times.

WSSV infection caused a decrease of shrimp THC in 12–24 h, raise a little at 36 h and dropped below control at 48 h (Fig. 6). The inhibition of miR-100 leads to a higher THC, at 36–48 h, the THC number was significantly higher than WSSV only group. Meanwhile, after *Vibrio alginolyticus* infection, the THC was higher than control at 12 h, decreased at 24 h, at 36 h, the THC raised significantly beyond the control group and reach a maximum value, decreased again to an even number as control. After the expression of miR-100 was inhibited, the THC increased at 12 h compared to Vibrio only group, at 24–48 h, the THC decreased. Both WSSV and Vibrio could trigger the self-destruction process of apoptosis or phagocytosis to eliminate pathogen. The increasing of THC after miR-100 inhibition suggesting a negative role of miR-100 in anti-virus process. On the contrary, the decrease of THC suggesting a positive role of miR-100 in anti-bacteria process.

### Influence of miR-100 knockdown on early-stage and late-stage apoptosis

**Figure 7:**
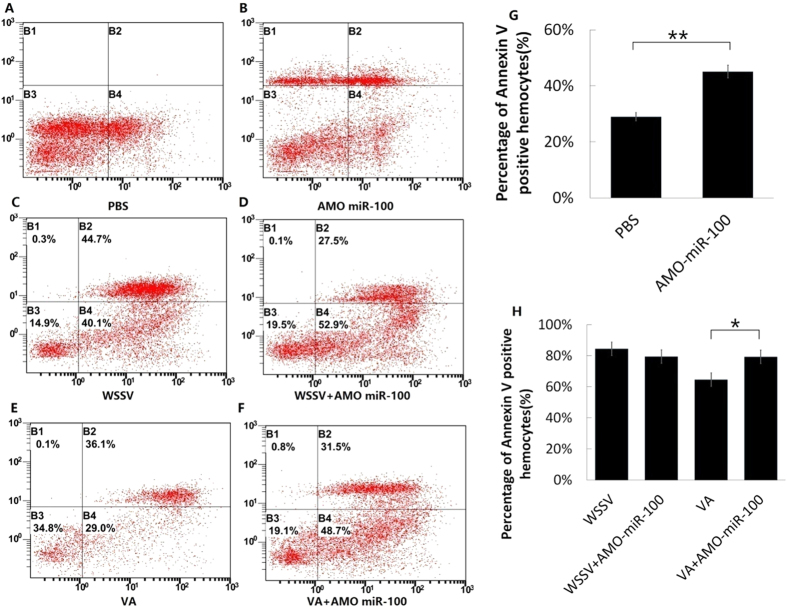
Effects of AMO-miR-100 interference on early- and late-stage of apoptosis. Shrimps were treated with a pathogen (WSSV or VA) only and with a combination of a pathogen and AMO-miR-100 (WSSV + AMO-miR-100 or VA + AMO-miR-100). PBS was used as a control. The shrimp hemocyte cells were stained with Annexin V and PI and detected by flow cytometry. B2 represents late-stage apoptosis, B4 denotes early-stage apoptosis, and B1 indicates healthy cells. The square graph depicts the apoptosis of hemocytes after the following treatments: (**A**) PBS-only treatment; (**B**) AMO-miR-100-only treatment; (**C**) WSSV-only treatment; (**D**) WSSV + AMO-miR-100 treatment; (**E**) VA-only treatment; (**F**) VA + AMO-miR-100 treatment. The percentages of Annexin V-positive cells; (**G**) PBS- and AMO-miR-100-only treatments; and (**H**) pathogen treatments are presented in the bar graphs, the asterisks marks indicate the significant differences (*P < 0.05, **P < 0.01).

Apoptosis assay of shrimp hemocytes with/without miR-100 knockdown was performed with detection by flow cytometry (FCM). In the PBS treatment, the percentage of Annexin V-positive hemocytes was 29.0% (Fig. 7A), which was slightly higher than the normal value of the control group (Fig. 7B) in a stabilized cell line of insect cells. We adjusted the pH of the EDTA anticoagulant, the PI dose, and the centrifugation parameters. Further, we minimized the progress time to avoid mechanical damage and reduce the early-stage apoptosis during the staining process. The vulnerability of shrimp hemocytes leads to the high extent of early-stage apoptosis and mechanical damage. Maximal efforts were exerted to minimize the damage and ensure the reliability of our results. The apoptosis of shrimp hemocytes increased substantially after their treatment with AMO-miR-100 for 36 h, showing that the knockdown of miR-100 would increase the apoptosis rate of shrimp hemocytes. Moreover, the apoptosis percentage of shrimp hemocytes treated with AMO-miR-100 was nearly two-fold higher than that of PBS treatment (Fig. 7G), indicating that the lack of miR-100 can induce apoptosis in shrimp hemocytes. These findings suggest an essential role of miR-100 and the considerable influence of its negative regulation on the apoptosis of shrimp hemocytes. The apoptosis percentage of WSSV (Fig. 7C,H) treatment was slightly higher (no significant difference) than WSSV + AMO-miR-100 treatment (Fig. 7D,H), while the apoptosis percentage of the *V. alginolyticus* treatment was significantly higher (*P* < 0.05) than that of the *V. alginolyticus* + AMO-miR-100 treatment (Fig. 7E,F and H). This result indicated that the negative regulation of miR-100 on apoptosis was more effective in *V. alginolyticus*-infected shrimp than in WSSV-infected shrimp.

### Influence of miR-100 knockdown on cell morphology and phagocytosis

**Figure 8:**
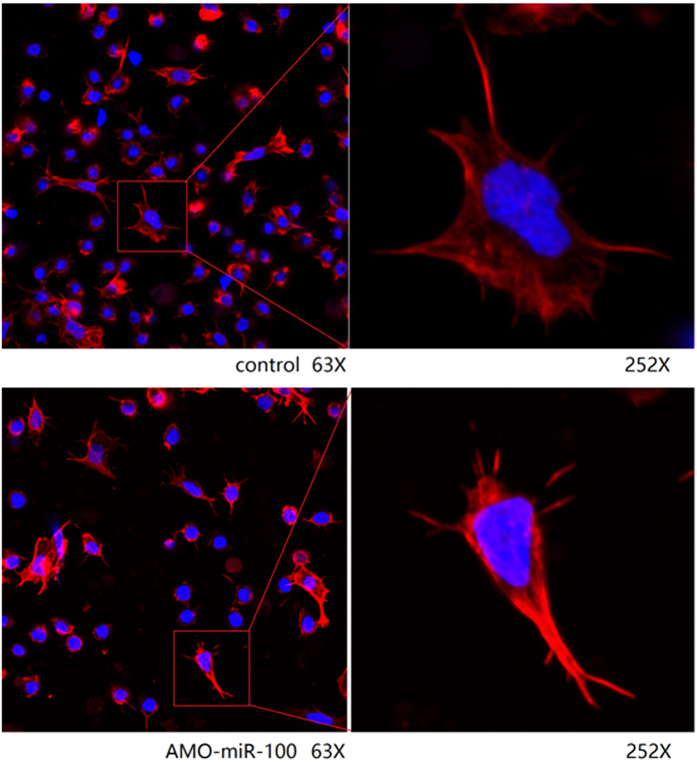
Confocal microscopy of shrimp hemocytes treated with AMO-miR-100. The filamentous actin was stained with rhodamine phalloidin and the cellular DNA with DAPI. Non-treated cells were used as controls. The amplification factor is 63 × (left) and 252 × (right).

**Figure 9:**
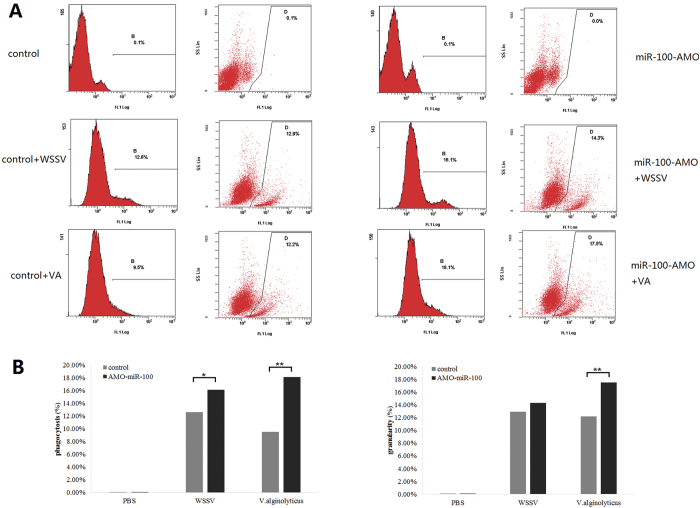
MiR-100-mediated regulation of phagocytosis in shrimp detected by flow cytometry. WSSV virions and *V. alginolyticus* were labeled with FITC. (**A**) The fluorescence peak area represented the fluorescence intensity in hemocytes, showing the percentage of pathogen-engulfed hemocytes, and the scatter plot represented the granularity of hemocytes, the increment of cell granularity suggested the ability of single cell to engulf pathogen. To show the influence of AMO-miR-100 more directly, the data above were transformed into bar chart (**B**).

To observe the cell morphology of hemocytes, they were stained and analyzed under a confocal microscope, and then the phagocytosis was detected by flow cytometry. The results showed that, in comparison with the control group, the cell extension of AMO-miR-100-treated hemocytes was not good as that of the control, and the cell morphology was abnormal, especially concerning the cytoskeleton (Fig. 8). Therefore, miR100 influenced the stability of cell skeleton to a certain extent. In addition, the phagocytosis rate (16.1%) in WSSV + AMO-miR100 was slightly higher than that of the WSSV group (12.6%) (Fig. 9). After the treatment with AMO-miR-100, the phagocytosis rate in the *V. alginolyticus* + AMO-miR-100 treatment (18.1%) was significantly higher (*P* < 0.01) than that in the *V. alginolyticus* group (9.5%). These data suggest that the knockdown of miR-100 would lead to more pronounced *V. alginolyticus* engulfing by phagocytes. This result indicates that miR-100 may negatively regulate the phagocytosis progress.

## Discussion

Increasing evidence indicates that miRNAs participate in almost every step of cellular processes and are often aberrantly expressed in human cancer^3^,^4^,^5^,^6^. Reportedly, miR-100 is involved in the tumorigenesis and tumor progression of several cancer types^16^,^17^,^18^,^19^,^20^,^21^,^22^. Moreover, shrimp miR-100 exerts important functions in the innate immunity and regulates the process of apoptosis in response to virus infections^23^,^25^. Through small RNA sequencing, miR-100 was found to be over-expressed after infection with *V. alginolyticus*^15^. Hence, our study contributes to elucidating the role of miR-100 in shrimp immune response to WSSV and *V. alginolyticus* infections.

In loss function experiment, we found there were two genes, among multiple major immune related genes, were influenced by the knock-down of miR-100. Hemocyanin, and Myosin. Previous research has shown that hemocyanin is an important respiratory protein which transports oxygen throughout the bodies of shrimps^26^ and might be involved in their resistance to pathogenic infections^27^,^28^,^29^,^30^. The data obtained in this study indicated that hemocyanin expression was regulated by miR-100. Myosin is a cytoskeleton protein which regulates multiple processes, such as material transport, muscle contraction, cell division and phagocytosis^31^,^32^. However, myosin is mostly known for its essential role in muscle construction and motility processes. In a previous study, the light chain of myosin was extracted from shrimp^32^. The findings of the investigation confirmed that shrimp myosin responded to virus invasion and was involved in the regulation of hemocytic phagocytosis. The roles of phagocytes and phagocytosis were crucial in the response of the innate immune system, and the down-regulation of myosin under AMO-miR-100 treatment suggested a potential influence of miR-100 on phagocytosis.

Recently, many studies have confirmed that miR-100 can regulate multiple genes to induce the cell apoptosis in many types of mammalian cell lines. There is a growing body of evidence that miR-100 inhibits the progression of apoptosis in human cancer cells through multiple pathways. Shrimp miR-100 was found to be an inhibitor in the apoptosis process of shrimp hemocytes in previous report^23^. Our research presented a similar conclusion, in contrast to the mechanism for control of apoptosis in human tumor cells, miR-100 negatively regulates the process of apoptosis in shrimp hemocytes. Our results revealed that WSSV infection would inhibit this negative regulation but *V. alginolyticus* infection would not.

To explore other effect of miR-100 on shrimp innate immunity, several functional parameters for evaluating the immune potential have been detected. In WSSV-infected shrimps, as the miR-100 expression was inhibited, the PO activity and SOD activity was significantly decreased. In *V. alginolyticus* infected shrimps, the PO activity was slightly increased at early stage, and SOD activity was relatively lower and showed a less activity increasing trend. The less effective function of PO and SOD implying a positive role of miR-100 in the anti-virus and anti-*Vibrio* respond. The THC post WSSV infection increased under miR-100 knock-down, while the THC post *V. alginolyticus* infection decreased. The same results were obtained in the apoptosis analyze. The mortality of *V. alginolyticus*-infected shrimps was also elevated. Meanwhile, the knockdown of miR-100 resulted in an increase in the phagocytosis rates of both WSSV-infected and *V. alginolyticus*-infected shrimps. This result indicated that miR-100 also inhibited the phagocytosis ability of shrimp hemocytes.

Synthesized into a conclusion, these findings indicate that shrimp miR-100 plays an essential role in the innate immune system of shrimp. miR-100 promotes the anti-*Vibrio* immune response of shrimp through regulating THC, apoptosis, phagocytosis, and PO activity. Furthermore, it exerts an influence on WSSV infection at a certain level via controlling SOD activity, hemocyte phagocytosis and PO activity.

## Methods

### Shrimps and tissue preparation

Total of healthy adult *M. japonicus* individuals were obtained from the seafood market of Hangzhou, Zhejiang, China. The hemocytes were collected from infected and uninfected shrimps. The samples were immediately used for RNA extraction to prevent total RNA degradation^15^.

### WSSV and *V. alginolyticus* preparation

WSSV (GenBank accession no. AF 332093.1) was purified and used in a challenge experiment as previously described^33^. *V. alginolyticus* was cultured and used to challenge the shrimps accord to a protocol reported earlier^34^.

### Sequencing of small RNAs

Total RNAs were extracted from the hemocytes of the infected and uninfected shrimps at 24 h and 48 h post-infection by using a miRNA isolation kit (Ambion, USA) in accordance with the manufacturer’s protocol. The next steps were performed following the guidelines outlined in a previously mentioned report^15^.

### Northern blotting of miRNAs

The small RNAs were extracted from shrimp hemocytes using mirVana miRNA isolation kit (Ambion, USA) according to the manufacturer’s manual. The concentration of the small RNA was measured using NanoDrop, and 3 mg small RNAs per sample were used for Northern blotting as previously described^15^.

### *In vitro* loss-of-function assay of shrimp miR-100

To silence the expression of miR-100(AACCCGTAGATCCGAACTTGTG), the anti-miRNA-100 oligonucleotide (AMO-miR-100) was injected into each shrimp at a dose of 0.15 mM/shrimp. As a control, the sequence of AMO-miR-100 was scrambled, and shown as AMO-miR-100-scramble. The AMO-miR-100-scramble (0.15 mM/shrimp) and phosphate buffered saline (PBS) were included in the injections. To avoid the degradation of AMO-miR-100 or AMO-miR-100-scramble, the oligonucleotides were sequentially injected into the same shrimp three times within a 24-h interval. Twenty-four hours after the last injection, the shrimp hemocytes from three random shrimp individuals were mixed and subjected to Northern bolt and phagocytosis assays. All the assays described above were biologically repeated three times.

### Quantitative real-time PCR

Total RNA of hemocytes was extracted by Easy spin tissue/cell RNA extra kit (Aidlab, China) following the protocol of manufactures. Experiments were performed in triplicates, and at least three shrimps were analyzed for each type of tissue. Less than 200 μg total RNA was used for cDNA synthesis by ReverTra Ace qPCR RT Master Mix with gDNA Remover Code: FSQ-301 (Toyobo, Japan). The cDNA was stored at −20 °C. A SYBR Green qRT-PCR assay was carried out in a Bio-Rad Two Color Real-Time PCR Detection System, and the data were calculated accord to the 2−ΔΔCT comparative CT method using Microsoft Office Excel 2007, with the amplification of GAPDH as an internal control. Designing and synthesizing of the qRT-PCR primers were entrusted to Generay Shanghai Company, based on the open read frame (ORF). The primer sequences for SYBR Green RT-PCR are shown in Table 1.

### Quantitative real-time PCR of miRNA

Total RNA of hemocytes was extracted by *mir*Vana^TM^ miRNA Isolation Kit (Thermo Fisher Scientific, USA) following the protocol of manufactures. Experiments were performed in triplicates, and at least three shrimps were analyzed for each type of tissue. Less than 200 μg total RNA was used for cDNA synthesis by TaqMan^TM^ MicroRNA Reverse Transcription Kit (Thermo Fisher Scientific, USA). The reverse transcription primer of miR-100 and real-time PCR primers of miR-100 and U6 were designed based on Stem-Loop miRNA primer design principle, the sequences were showed in Table 1. The cDNA was stored at −20 °C. A SYBR Green qRT-PCR assay was carried out in a Bio-Rad Two Color Real-Time PCR Detection System, and the data were calculated accord to the 2−ΔΔCT comparative CT method using Microsoft Office Excel 2007, with the amplification of U6 as an internal control.

### Shrimp mortality analysis

Shrimp (over 20 individuals) were injected intramuscularly with AMO-miR-100 in the lateral area of the fourth abdominal segment. Twelve hours later, the treated shrimps were injected with 0.1 mL of filtrate (10^5^ WSSV copies/mL) containing AMO-miR-100 by using a syringe with a 29-gauge needle. As controls, shrimps (over 20 individuals) were injected with PBS only or WSSV only. After the last injection, the mortality of shrimps was monitored every day.

### Total Hemocyte Count analysis

Approximately 1.0–1.5 mL shrimp hemolymph was collected using an anticoagulant soaked syringe, together with equal volume anticoagulant (450 mM NaCl, 10 mM EDTA-Na2, 10 mM HEPES, pH 7.3, 850 mOsm/kg)^35^. The mixture was kept on ice. Add 1/3 volume 4% paraformaldehyde to immobilize the hemocyte, and then count THC using hemocytometer by phase contrast microscope (Nikon, ECLIPSE Ti-S, Japan). Based on the calculate method of the hemocytometer plate, the THC of each group presented the total hemocyte count in 1 mL hemolymph.

### Phagocyte rate counting by flow cytometry

After purchase, the shrimps were stored in ice-cold sea water until use. Then, the shrimps were sterilized with 70% alcohol, and hemolymph was withdrawn and mixed 1:1 in a disposable syringe containing 20 mM EDTA. Next, to collect hemolymph cells, the samples were centrifuged at 2000 rpm x 10 min at 4 °C. The cells were gently suspended thoroughly with high salty PBS under aseptic conditions, the cell count number was determined by an auto cell counter, and their density was adjusted to 3–5 × 10^6^. The separate cells were divided into three equal tubes used as a control, WSSV, and VA group, respectively. FITC-labeled WSSV or VA was added in the hemolymph cells accord to the procedure described in a previous report^36^. Further, the samples were set still for 20 min, after which, the extra pathogens were washed with PBS 3 times, and 500 μL of PBS with 1% PFA (paraformaldehyde) was added for conducting the flow cytometry test.

### Phenol oxidase (PO) activity analysis

To determine the influence of miR-100 on shrimp PO activity, healthy shrimps were divided into five groups: three groups were treated with PBS, WSSV, and *V. alginolyticus*, respectively, while the other two groups were treated with a mixture of AMO-miR-100 and pathogens (WSSV + AMO-miR-100 and *V. alginolyticus* + AMO-miR-100, respectively. At different time points post-injection, the hemolymph of each group was obtained and centrifuged at 300 g for 10 min to separate the hemocyte cells and the serum. Equal volumes of serum (10 µL) of each group were incubated with 10 uL of L-DOPA in 80 µL saline for 15 min at room temperature. The incubated serums were applied to measure the PO activity using a BioRad spectrophotometer with detection at a wavelength of 490 nm.

### Superoxide Dismutase(SOD) activity analysis

Shrimp hemolymph of each group was extracted for SOD extraction. 100ul hemolymph was homogeneous mixed with 0.5 ml phosphate buffer (PB, 50 mM, pH7.8), then centrifuged at 5724 × g for 5 min, 4 °C. The supernatant was separated into a clean new tube, followed by 65 °C heating for 5 min, then centrifuge for 5 min. Separated the new supernatant, and store at −20 °C for further assay. To avoid protein degeneration, samples were kept on ice always.

SOD activity was determined using Nitro blue tetrazolium method (NBT) created by Beauchamp^37^ and improved by Bewley^38^ In this method, 0.05 ml pre-extracted hemolymph SOD sample of each treatment was added into 0.95 ml reaction mixture (0.1 mM EDTA, 13 μM methionine, 0.75 mM NBT, and 20 μM riboflavin, in 50 mM PB, pH 7.8) in a pellucid clean 1.5 ml tube. To activate the photo-reduction of riboflavin, put the tubes under 4000Lx fluorescent light for 1–2 min, or until OD560 of control tube (replace SOD sample with 50 μl PB) reached 0.2–0.25.

The specific SOD activity unit was defined as the enzyme amount that can induce half reaction rate of the initial reaction rate (without enzyme). The formula below was applied to calculate SOD activity units. SOD unit of PBS groups were used as an index to compare the different effect of treatments on the SOD activity. The results were expressed as Relative SOD activity.

SOD activity unit = (OD560control-OD560sample)/50% OD560control * dilution ratio

### Apoptosis analysis with Annexin V

Apoptosis assay of shrimps with/without miR-100 knockdown was conducted with Annexin V (Invitrogen, USA) accord to the manufacturer’s protocol. Shrimp hemolymph from the treatment groups were harvested in 1:1 with 20 mM EDTA, centrifuged at 2000 rpm x 10 min to collect hemocytes, and washed once in pre-cold high-salt PBS. The hemocytes were gently suspended in 1 × Annexin-Binding buffer. Then, 5 μL of Alexa Flour 488 Annexin V and 1 μg/mL of PI (propidium iodide) were added. The samples were incubated at room temperature for 15 min or at 4 °C for 30 min. Next, to end the reaction, 400 μL of 1 × Annexin-binding buffer was added into the samples. Finally, the fluorescence emission of the stained samples was examined by flow cytometry at wavelengths of 530 nm and 575 nm.

### Statistical analysis

The data from three independent experiments were analyzed by one-way analysis of variance (ANOVA) to calculate the mean and standard deviation of the triplicate assays. The differences between the different treatments were analyzed by *t*-test.

## Additional Information

**How to cite this article:** Zhi, W. and Fei, Z. MicroRNA-100 is involved in shrimp immune response to white spot syndrome virus (WSSV) and *Vibrio alginolyticus* infection. *Sci. Rep.* **7**, 42334; doi: 10.1038/srep42334 (2017).

**Publisher's note:** Springer Nature remains neutral with regard to jurisdictional claims in published maps and institutional affiliations.

## Figures and Tables

**Table 1:** Primers used in this study.

Primer Name	Primer Sequence (5′ to 3′)	Used for
U6	GGGCCATGCTAATCTTCTCTGTATCGTT	Northern Blot probe
miR-100	CACAAGTTCGGATCTACGGGTT	
AMO-miR-100	TTCGGATCTACgGGtT	miRNA Knockdown
AMO-miR-100 scrambled	GGTCTCATATGcTTgG	
miR-100 RT	GTCGTATCCAGTGCAGGGTCCGAGGTCACTGGATACGACCACAAGT	MiRNA Reverse transcription
miR-100-F	CGCCGAACCCGTAGATCCG	miRNA real-time PCR
miR-100-R	TGCAGGGTCCGAGGTCACTG	miRNA real-time PCR
U6 RT	GTCGTATCCAGTGCAGGGTCCGAGGTCACTGGATACGACCTCACTT	MiRNA Reverse transcription
U6-F	TTCACGAATTTGCGTGTCAT	miRNA real-time PCR
U6-R	CGCTTCGGCAGCACATATAC	miRNA real-time PCR
GAPDH-F	GGTGCCGAGTACATCGTTGAGTC	Real-time PCR
GAPDH-R	GGCAGTTGGTAGTGCAAGAGGC	
Rab7-F	TCATTAGGTGTTGCATTTTATCGC	
Rab7-R	AGGCTTGAATTAGGAACTCGTC	
*hemocyanin*-F	AACCCTGAACAAAGAGTTGCCTAT	
*hemocyanin*-R	AACGGACGGTAAGTTGATGATGT	
IMD-F	ATTCATCCGTCTACCTCCCTACA	
IMD-R	GAGCTGAGTCTGTCTTAATGTTATCC	
*myosin* -F	GCCCAGGTCAAGAAGGACAAGGA	
*myosin* -R	AAGACGCTCACCAAGGGACAGGA	
*p*53-F	TTCCTGCCTGGCTGACTCTA	
*p*53-R	CACCCAATCTTCCAACATCACAT	
MAPK-F	CGCATCACTGTTGAGGAGG	
MAPK-R	GCAGGTCATCAAGTTCCATCT	
*pro*PO-F	TTCTACCGCTGGCATAAGTTTGT	
*pro*PO-R	TATCTGCCTCGTCGTTCCTCAC	
Rab7-F	TCATTAGGTGTTGCATTTTATCGC	
Rab7-R	AGGCTTGAATTAGGAACTCGTC	
Rho-F	GTGATGGTGCCTGTGGTAAA	
Rho-R	GCCTCAATCTGTCATAGTCCTC	
L-lectin-F	ATGTTATGCCATCTGCCTCGTATTT	
L-lectin-R	CTTTCGCTGCTGCTCTTTCTGTT	
STAT-F	TGGCAGGATGGATAGAAGACAAG	
STAT-R	TGAATAAGCTGGGATACGAGGGA	
TNF-F	ACAGACGGTCCAGAGTCCCAAAG	
TNF-R	GCGACGAAGTGAGCCACAGTAA	

## References

[b1] Ambros V. microRNAs: Tiny Regulators with Great Potential. Cell 107, 823–826 (2001). 11779458 10.1016/s0092-8674(01)00616-x

[b2] Schmiedel J. M. . microRNA control of protein expression noise. Science 348, 128–132 (2015). 25838385 10.1126/science.aaa1738

[b3] Xiao C. & Rajewsky K. microRNA control in the immune system: basic principles. Cell 136, 26–36 (2009). 19135886 10.1016/j.cell.2008.12.027

[b4] Wienholds E. & Plasterk R. H. A. MicroRNA expression in zebrafish embryonic development. Science 309, 310–311 (2005). 15919954 10.1126/science.1114519

[b5] Calin G. A. & Croce C. M. MicroRNA-cancer connection: the beginning of a new tale. Cancer Res 66, 7390−7394 (2006). 16885332 10.1158/0008-5472.CAN-06-0800

[b6] Yu S. L. . MicroRNA signature predicts survival and relapse in lung cancer. Cancer Cell 13, 48–57 (2008). 18167339 10.1016/j.ccr.2007.12.008

[b7] Huang J. Y. . MicroRNA-130a can inhibit hepatitis b virus replication via targeting PGC1α and PPARγ. RNA 21, 385–400 (2015). 25595716 10.1261/rna.048744.114 PMC4338335

[b8] Kumar S. . Overexpression of microRNA-30a inhibits hepatitis b virus x protein-induced autophagosome formation in hepatic cells. FEBS J 282, 1152–1163 (2015). 25620738 10.1111/febs.13209

[b9] Mukherjee A., Di B. A. & Ray R. B. Hepatitis c virus-mediated enhancement of microRNA mir-373 impairs the JAK/STAT signaling pathway. J Virol 89, 3356–3365 (2015). 25589644 10.1128/JVI.03085-14 PMC4337546

[b10] Motavaf M., Safari S. & Alavian S. M. Targeting microRNA-122: walking on cutting edge of hepatitis C virus infection therapy. Acta Virol 58, 301–308 (2014). 25518710 10.4149/av_2014_04_301

[b11] Wen W. . Cellular microRNA-mir-548g-3p modulates the replication of dengue virus. J Infection 70, 631–640 (2015). 10.1016/j.jinf.2014.12.001 25499200

[b12] Patel P. . The microRNA miR-29a is associated with human immunodeficiency virus latency. Retrovirology 11, 1–5 (2013). 10.1186/s12977-014-0108-6 PMC4269869 25486977

[b13] Ruan L. . Isolation and identification of novel microRNAs from *Marsupenaeus japonicus*. Fish Shellfish Immunol 31, 334–340 (2011). 21658454 10.1016/j.fsi.2011.05.023

[b14] Huang T., Xu D. & Zhang X. Characterization of host microRNAs that respond to DNA virus infection in a crustacean. BMC Genomics 13, 159 (2012). 22545795 10.1186/1471-2164-13-159 PMC3411463

[b15] Zhu F., Wang Z. & Sun B. Differential expression of microRNAs in shrimp *Marsupenaeus japonicus* in response to *Vibrio alginolyticus* infection. Dev Comp Immunol 55, 76–79 (2015). 26483347 10.1016/j.dci.2015.10.012

[b16] Peng D. X., Luo M., Qiu L. W., He Y. L. & Wang X. F. Prognostic implications of microRNA-100 and its functional roles in human epithelial ovarian cancer. Oncol Rep 27, 1238–1244 (2012). 22246341 10.3892/or.2012.1625 PMC3583406

[b17] Liu J. . MicroRNA-100 is a potential molecular marker of non-small cell lung cancer and functions as a tumor suppressor by targeting polo-like kinase 1. BMC Cancer 12, 1–11 (2012). 23151088 10.1186/1471-2407-12-519 PMC3521172

[b18] Zhou M. K., Liu X. J., Zhao Z. G. & Cheng Y. M. MicroRNA-100 functions as a tumor suppressor by inhibiting Lgr5 expression in colon cancer cells. Mol Med Rep 11, 2947–2952 (2014). 25483280 10.3892/mmr.2014.3052

[b19] Chen P., Zhao X. & Liang M. Downregulation of microRNA-100 correlates with tumor progression and poor prognosis in hepatocellular carcinoma. Mol Cell Biochem 383, 49–58 (2013). 23842624 10.1007/s11010-013-1753-0

[b20] Alrfaei B. M., Vemuganti R. & Kuo J. S. MicroRNA-100 targets SMRT/NCOR2, reduces proliferation, and improves survival in glioblastoma animal models. Plos One 8, e80865 (2013). 24244722 10.1371/journal.pone.0080865 PMC3828259

[b21] Huang J., Gao K., Lin J. & Wang Q. MicroRNA-100 inhibits osteosarcoma cell proliferation by targeting Cyr61. Tumour Biol 35, 1095–1100 (2014). 24317814 10.1007/s13277-013-1146-8

[b22] Peng H. . MicroRNA-100 regulates SW620 colorectal cancer cell proliferation and invasion by targeting RAP1B. Oncol Rep 31, 2055–2062 (2014). 24626817 10.3892/or.2014.3075

[b23] Yang L., Yang G. & Zhang X. The miR-100-mediated pathway regulates apoptosis against virus infection in shrimp. Fish Shellfish Immunol 40, 146–153 (2014). 24972342 10.1016/j.fsi.2014.06.019

[b24] Zhu F. & Zhang X. Protection of shrimp against white spot syndrome virus (WSSV) with β-1,3-d-glucan-encapsulated VP28-siRNA particles. Mar. Biotechnol 14, 63–68 (2012). 21590271 10.1007/s10126-011-9387-2 PMC7087676

[b25] Beltramini M. . Quaternary structure and functional properties of *Penaeus monodon*, hemocyanin. FEBS J 272, 2060–2075 (2005). 15819896 10.1111/j.1742-4658.2005.04634.x

[b26] Lu X. . Cloning and characterization of a novel hemocyanin variant LvHMCV4 from shrimp *Litopenaeus vannamei*. Fish Shellfish Immunol 46, 398–405 (2015). 26115633 10.1016/j.fsi.2015.06.022

[b27] Qiu C. . Molecular cloning of hemocyanin cDNA from *Fenneropenaeus chinensis* and antimicrobial analysis of two C-terminal fragments. Mar Biotechnol 16, 46–53 (2014). 23887674 10.1007/s10126-013-9519-y

[b28] Zhang X. & Huang C. Q. Antiviral properties of hemocyanin isolated from shrimp *Penaeus monodon*. Antiviral Res 61, 93–99 (2004). 14670582 10.1016/j.antiviral.2003.08.019

[b29] Lei K. . Difference between hemocyanin subunits from shrimp *Penaeus japonicus* in anti-WSSV defense. Dev Comp Immunol 32, 808–813 (2008). 18234332 10.1016/j.dci.2007.11.010

[b30] Liu W., Han F. & Zhang X. Ran GTPase regulates hemocytic phagocytosis of shrimp by interaction with myosin. J Proteome Res 8, 1198–1206 (2009). 19166347 10.1021/pr800840x

[b31] Han F., Wang Z. & Wang X. Characterization of myosin light chain in shrimp hemocytic phagocytosis. Fish Shellfish Immunol 29, 875–883 (2010). 20691789 10.1016/j.fsi.2010.07.030

[b32] Taengchaiyaphum S. . Phosphorylation is required for myosin regulatory light chain (PmMRLC) to control yellow head virus infection in shrimp hemocytes. Fish Shellfish Immunol 34, 1042–1049 (2013). 23337109 10.1016/j.fsi.2012.12.022

[b33] Huang M., Liu Y., Xie C. & Wang W. N. Lvdj-1, plays an important role in resistance against *Vibrio alginolyticus*. In Litopenaeus vannamei. Fish Shellfish Immunol 44, 180–6 (2015). 25703712 10.1016/j.fsi.2015.02.022

[b34] Zhu F. & Zhang X. The Wnt signaling pathway is involved in the regulation of phagocytosis of virus in *Drosophila*. Sci Rep 3, 2069–2069 (2013). 23797713 10.1038/srep02069 PMC3691566

[b35] Cordova A. I., Hernandez-Saavedra N. Y., De P. R. . Generation of superoxide anion and SOD activity in haemocytes and muscle of American white shrimp (Litopenaeus vannamei) as a response to beta-glucan and sulphated polysaccharide. Fish Shellfish Immunol 4, 353–366 (2002). 10.1006/fsim.2001.0377 12049170

[b36] Yang G., Yang L., Zhao Z., Wang J. & Zhang X. Signature miRNAs involved in the innate immunity of invertebrates. Plos One 7, e39015 (2012). 22723921 10.1371/journal.pone.0039015 PMC3378607

[b37] Beauchamp C. & Fridovich I. Superoxide dismutase: Improved assays and an assay applicable to acrylamide gels ☆. Anal. Chem 1, 276–287 (1971). 10.1016/0003-2697(71)90370-8 4943714

[b38] Bewley J. D. Physiological Aspects of Desiccation Tolerance. Boundary-Layer Meteorology 39, 315–332 (2003).

